# The complete mitochondrial genome of freshwater gammarid *Gammarus nipponensis* (Crustacea: Amphipoda: Gammaridae)

**DOI:** 10.1080/23802359.2024.2335990

**Published:** 2024-04-03

**Authors:** Joana Joy de la Cruz-Huervana, Norio Miyamoto, Yuichi Kano, Norio Onikura, Yoshihisa Kurita

**Affiliations:** a Fishery Research Laboratory, Kyushu University, Japan; b Aquaculture Department, Southeast Asian Fisheries Development Center, Philippines; c X-STAR, Japan Agency for Marine-Earth Science and Technology (JAMSTEC), Yokosuka, Japan; d Kyushu University, Fukuoka, Japan; e Kyushu Open University, Fukuoka, Japan

**Keywords:** Complete mitogenome, amphipods, Gammaroidea, phylogeny

## Abstract

This study presents the complete mitochondrial genome sequence of *Gammarus nipponensis*, a freshwater crustacean found in the western regions of Honshu, Shikoku and Kyushu in Japan. The entire genome is 16,429 bp in length, encoding a standard set of 13 protein-coding genes, two ribosomal RNA genes and 22 transfer RNA genes, as well as the putative control regions. The mitochondrial genome of *G. nipponensis* is characterized by a high concentration of A and T nucleotides (67.1%). Notably, the mitogenome contains long TATTTTA repeats in the control region 2 at 686 bp long. This newly available genome information will be useful for studying the evolutionary relationships within the genus *Gammarus* and for understanding diversification among *G. nipponensis* populations.

## Introduction

The genus *Gammarus* Fabricius 1775, is considered one of the most diverse genera of crustaceans (Väinölä et al. 2008). In Japan, three *Gammarus* species have been recorded: *Gammarus nipponensis* Uéno 1940, *Gammarus koreanus* Uéno 1940 and *Gammarus mukudai* Tomikawa et al. 2014. Among these, *G. nipponensis* has the widest distribution, inhabiting western Honshu, Shikoku and Kyushu (Tomikawa 2017). These *Gammarus* species are exclusively found in freshwater habitats which limits dispersal. Moreover, the life cycle of gammarids involves direct development, where the fertilized eggs are brooded until the juvenile stage (Hyne 2011; Bastos-Pereira and Bueno 2016). Given these characteristics, *G. nipponensis* can be used to examine the effect of habitat fragmentation on the degree of genetic variation within and among their populations.

Previous study on the phylogenetics of *G. nipponensis* relied on *cox1* and *28s* rRNA genes (Tomikawa et al. 2014) and the classification and phylogenetic status of genus *Gammarus* remains problematic (Hou et al. 2007; 2014, Hou and Sket 2016, Macher et al. 2017). As a result, the full mitochondrial genome of *G. nipponensis* can give useful resources for additional phylogeographic information. Furthermore, this is the first complete mitochondrial genome for genus *Gammarus* in Japan.

## Materials and methods

**Figure 1.**
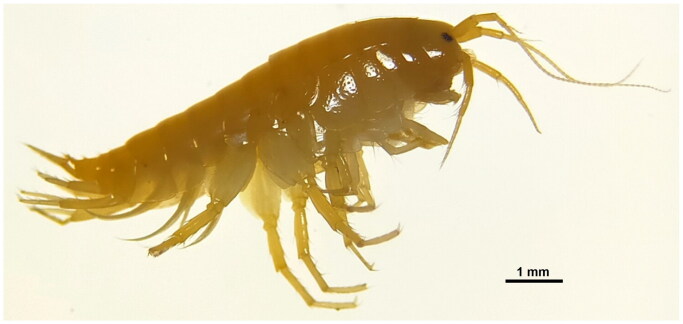
Freshly preserved specimen of *Gammarus nipponensis* (photo credits: J.D. Huervana).

The specimen of *G. nipponensis* (Figure 1) was obtained from Kuro River in Asakura, Fukuoka (33°23'56.8"N, 130°48'19.6"E) and immediately preserved in 95% ethanol. The species identification was based on the morphological characters as described in Tomikawa et al. (2014). According to the reference, the accessory flagellum is present on antenna 1, and the posterior margins of peduncular articles 4 and 5 in antenna 2 have 6 and 7 clusters of long setae, respectively.

This specimen was stored in Kyushu University and registered in the ffish.asia database (https://ffish.asia/?page=specimen&pid=53448; contact person: Y. Kurita, kurita.yoshihisa.070@m.kyushu-u.ac.jp) under specimen ID number QUYK-10765. Total genomic DNA was extracted from the pereopod muscles using the Quick DNA^TM^ Miniprep Plus Kit (Zymo Research, USA) following the manufacturer’s protocol.

The extracted DNA was submitted to Bioengineering Lab. Co., Ltd. (Sagamihara, Japan) for library construction and sequencing. Library preparation was done according to MGIEasy FS DNA Library Prep Set manual, and high-throughput DNA sequencing was performed using the DNBSEQ-G400 system (MGI Tech, Shenzhen, China). Raw reads (25,350,425 pairs of 200 bp) from the sequencing process were filtered using Sickle ver. 1.33 (Joshi and Fass 2011) to remove adapter sequences and reads with quality score less than 30. The resulting high-quality reads were assembled using metamode of SPAdes ver. 3.14.0 (Nurk et al. 2017), then annotated using the MITOS web server (Bernt et al. 2013) and sequentially manually corrected to improve accuracy. Results of the short read assembly revealed the presence of tandem repeats of TATTTTA in the mitochondrial genome of this species. PCR was conducted across the repeats using primers (Gnip CR2F: 5′-GTGATTACAATAACATTTGGCGGC-3′ and Gnip CR2R: 5′-GCTATAAGGCTGACCTCAAGGC-3′) and showed that the length of repeats was approximately 650 bp. Since its precise length could not be determined by Sanger sequencing, the number of repeats was confirmed using MinION sequencer (Oxford Nanopore Technologies) equipped with a FLO-MIN106D flow cell and SQK-RAD004 kit. The long reads were assembled with Flye ver. 2.9.1 (Kolmogorov et al. 2019), then the assembled contig was polished with Pilon ver. 1.22 using the short reads (Walker et al. 2014).

The read coverage depth obtained for the assembled mitogenome generated in DNBSEQ short reads was about 400x, with the lowest coverage depth (1x) at the repetitive region of CR2 (Figure S1). Improved coverage depth (∼1000x) in CR2 was achieved from Oxford Nanopore long reads, and the alignment in CR2 (Figure S2) suggests the reliability of the final assembly. Read coverage depth and circular map of the mitochondrial genome was visualized using the integrative genomics viewer (Thorvaldsdóttir et al. 2013) and Proksee server at https://proksee.ca/projects (Grant et al. 2023), respectively. The annotated sequence was submitted to NCBI GenBank with accession number OR268765.2.

Phylogenetic analysis was performed using nucleotide sequences from 13 protein-coding genes (PCG), encompassing all species within Gammaroidea (Taxonomy ID: 44329) available in the NCBI database. Two Lysianassidira species, *Alicella gigantea* Chevreux 1899 and *Eurythenes maldoror* D’Udekem d’Acoz and Havermans 2015, were selected as outgroups due to their sister relationship with Gammaroidea (Macher et al. 2023). The nucleotide sequences of each gene were aligned using the “auto” strategy in MAFFT ver. 7 (Katoh et al. 2019) and manually trimmed the ends. Phylogenetic trees were then generated using maximum likelihood method with the concatenated dataset, employing 10,000 ultrafast bootstrap replicates in IQ-TREE ver. 1.6.12 (Nguyen et al. 2015). To determine the best-fit substitution model, ModelFinder in IQ-TREE was employed under the Bayesian Information Criterion (Kalyaanamoorthy et al. 2017). The selected model was GTR + F + I + G4.

## Results

**Figure 2.**
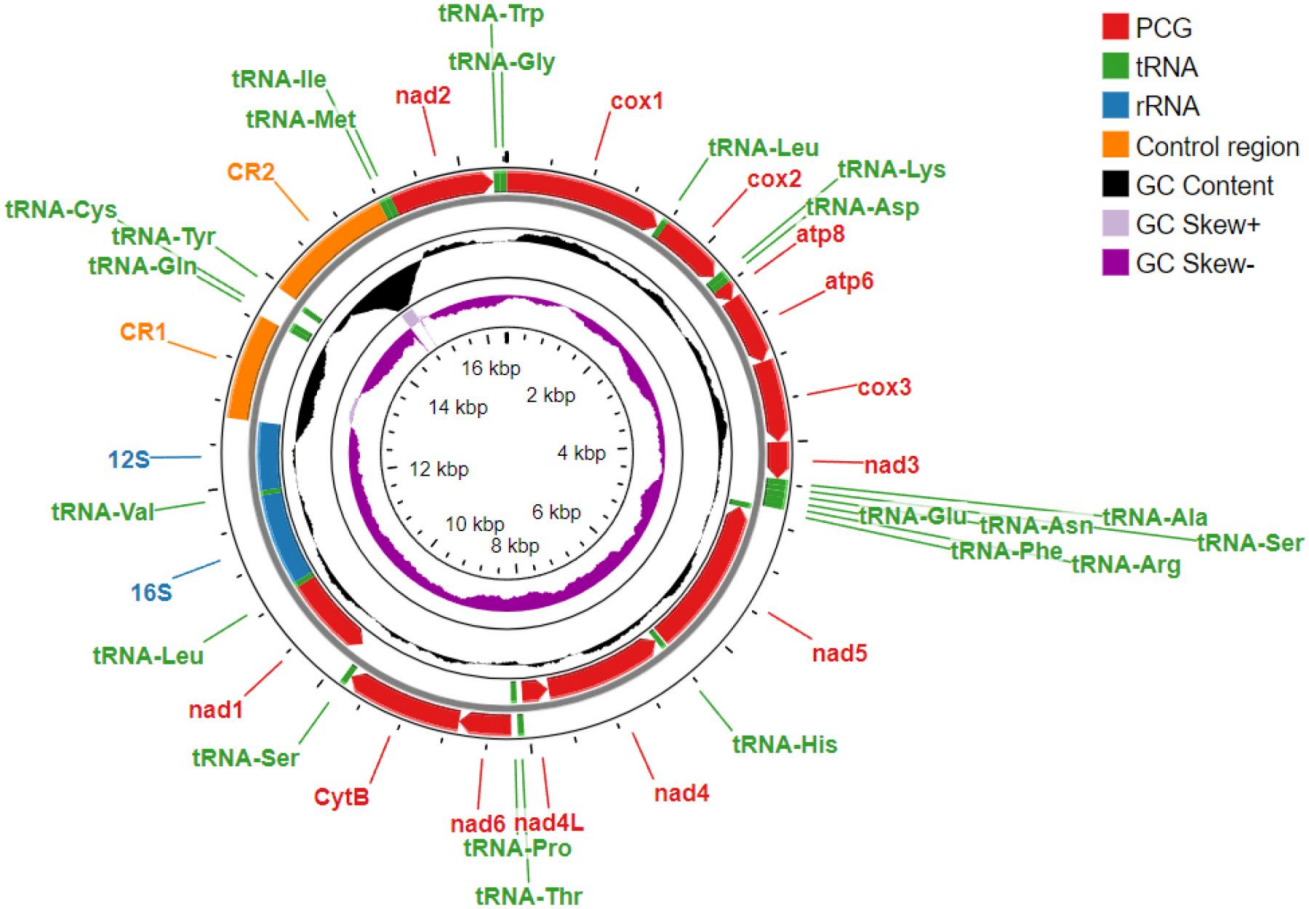
Mitochondrial genome map of *Gammarus nipponensis* showing the arrangement of PCGs, tRNAs, rRNAs and putative control regions.

The mitochondrial genome of *G. nipponensis* is 16,429 bp in length and contains 13 PCG (*cox1-3*, *nad1-6*, *nad4L*, *CytB*, *atp6* and *atp8*), two ribosomal RNAs (*16S* and *12S*), 22 transfer RNAs (two for Leu and Ser and one for each amino acid) and two putative control regions (CR) (Figure 2). The CR in *G. nipponensis* are found in two positions: CR1 – located after the *12S* gene and CR2 – situated between tRNA-*Tyr* and tRNA-*Ile*, with a combined length of about 2200 bp. Interestingly, the mitochondrial genome of *G. nipponensis* contained 98 TATTTTA repeats in CR2. Among the genes, nine of the 13 PCG and 14 of the 22 tRNAs are encoded by the forward (+) strand, while the remaining genes are encoded by the reverse (-) strand.

Nucleotide composition of the *G. nipponensis* mitogenome is AT-rich at 67.1% (A:31.7%, C:21.8%, G:11.1%, T:35.4%) and the overall AT-skew and GC-skew are −0.055 and −0.325, respectively. The PCGs make up 11,010 bp (67.13%) and encode 3659 amino acids.

**Figure 3.**
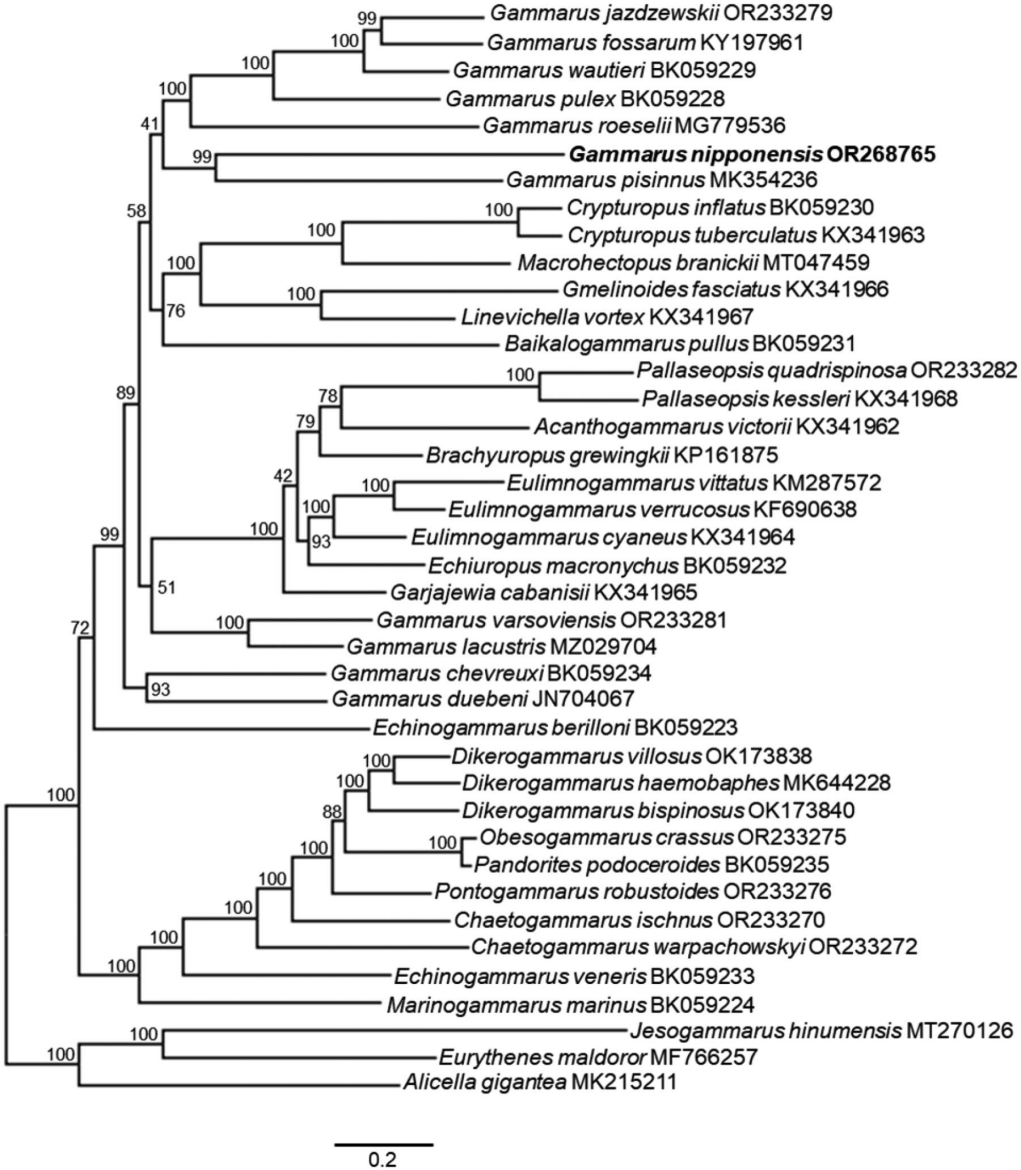
The maximum likelihood phylogenetic tree based on the sequence alignment of 13 protein-coding genes of *Gammarus nipponensis* and amphipods under superfamily Gammaroidea. Numbers on each node indicate the bootstrap support (%) values and scale bar signifies nucleotide sequence difference. *Alicella gigantea* and *Eurythenes maldoror* are used as outgroup. The following sequences were used: OR233279, OR233282, OR233281, OR233275, OR233276, OR233270, OR233272 (Macher et al. 2023); KY197961 (Macher et al. 2017); BK05230, BK059231, BK059229, BK059228, BK059232, BK059234, BK059223, OK173838, OK173840, BK059235, BK059233, BK059224 (Mamos et al. 2021); MG779536 (Cormier et al. 2018); OR268765 (this study); MK354236 (Sun et al. 2020); KX341963, KX341966, KX341967, KX341964, KX341965, KX341968, KX341962 (Romanova et al. 2016a); MT047459 (Romanova et al. 2021); KP161875 (Romanova et al. 2016b); KM287572 (Romanova et al. 2016c); KF690638 (Rivarola-Duarte et al. 2014); MZ029704 (Li et al. 2021); JN704067 (Krebes and Bastrop 2012); MK644228 (Bojko 2020), MT270126 (Lee et al. 2020), MF766257 (unpublished) and MK215211 (Li et al. 2019).

Molecular phylogenetic analysis showed that the mitochondrial genome sequence assembled in this study is positioned within the superfamily Gammaroidea with close affinity to *Gammarus pisinnus* (Figure 3).

## Discussion and conclusion

Mitochondrial genes of *G. nipponensis* are encoded similar to the putative ancestral pancrustacean ground pattern, however, there are differences in the order of tRNAs (Ser, Glu, Arg and Gly) and the occurrence of dual CR. Translocation of tRNAs is commonly observed in gammarids (Krebes and Bastrop 2012; Cormier et al. 2018; Mamos et al. 2021). Two CRs are also reported in *G. roeselii*, but these regions displayed nearly identical sequences unlike in our study species (Cormier et al. 2018). Another interesting feature of *G. nipponensis* mitogenome is the presence of tandem repeats within the CR. Although tandem repeats are also found in *Gammarus duebeni*, these are less abundant comprising six imperfect tandem repeats at 84 to 97 bp long, in contrast to those in *G. nipponensis* (Krebes and Bastrop 2012). Phylogenetic analysis suggested that *G. nipponensis* is closely related to *G. pisinnus* both of which are native to East Asia (Sun et al. 2020). Previous studies (Sun et al. 2020; Mamos et al. 2021; Macher et al. 2023) have highlighted the need for additional information on mitogenome sequences of gammarids for their more detailed phylogenetic relationship. Thus, the complete mitogenome of *G. nipponensis* contributes to such investigations. Also, the information from this study may lead to the development of improved tools for phylogenetic analysis among the populations of *G. nipponensis*.

## Supplementary Material

Supplemental Material

## Data Availability

The genome sequence data corresponding to this study are available in GenBank at https://www.ncbi.nlm.nih.gov with accession number: OR268765.2. The associated BioProject, SRAs and Bio-Sample number are PRJNA999456, SRR25443940, SRR26250192 and SAMN36734956, respectively. The sequence read SRR25443940 corresponds to DNBSEQ data, while SRR26250192 corresponds to Oxford Nanopore data.
