# Supplementary material for: The complete mitochondrial genome of freshwater gammarid Gammarus nipponensis (Crustacea: Amphipoda: Gammaridae)
Source: Mitochondrial DNA B Resour. 2024 Apr 3;9(4):447–51. doi: 10.1080/23802359.2024.2335990 (PMC10993753; doi:10.1080/23802359.2024.2335990)
Supplement: Supplemental Material [file TMDN_A_2335990_SM9284.docx]

**The complete mitochondrial genome of freshwater gammarid *Gammarus nipponensis* (Crustacea: Amphipoda: Gammaridae)**

**Supplementary files**


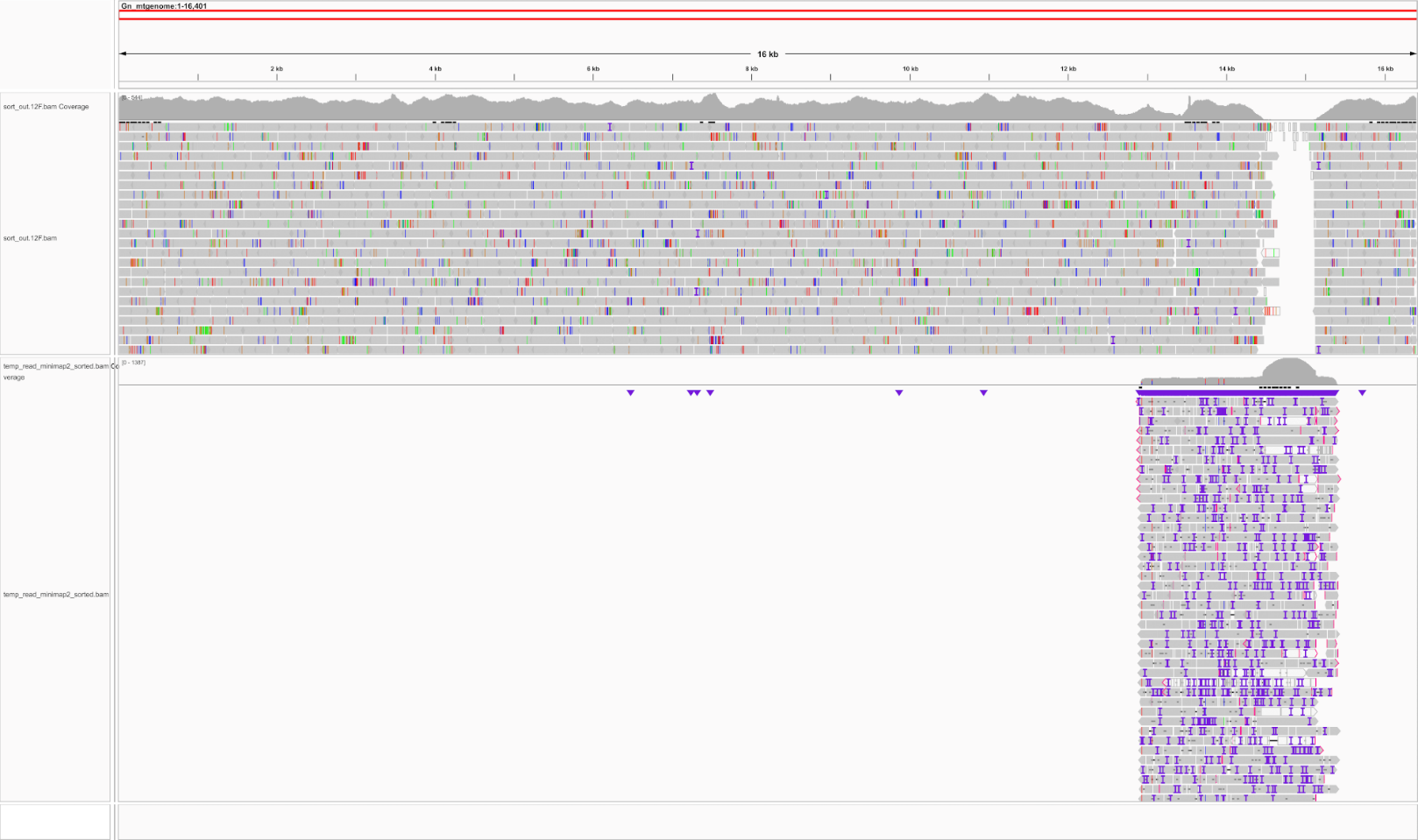


**Figure S1.** The read coverage data of *Gammarus nipponensis* mitochondrial genome is presented. The upper panel shows the coverage depth of DNBSEQ short reads (~400x), exhibiting a substantial decrease at the repetitive region of control region 2 at 1x. While, the lower panel shows the coverage depth of Oxford Nanopore long reads (>1000x), revealing an increase in coverage within the control region 2. This illustration was generated using the integrative genomics viewer (Thorvaldsdóttir et al. 2013).


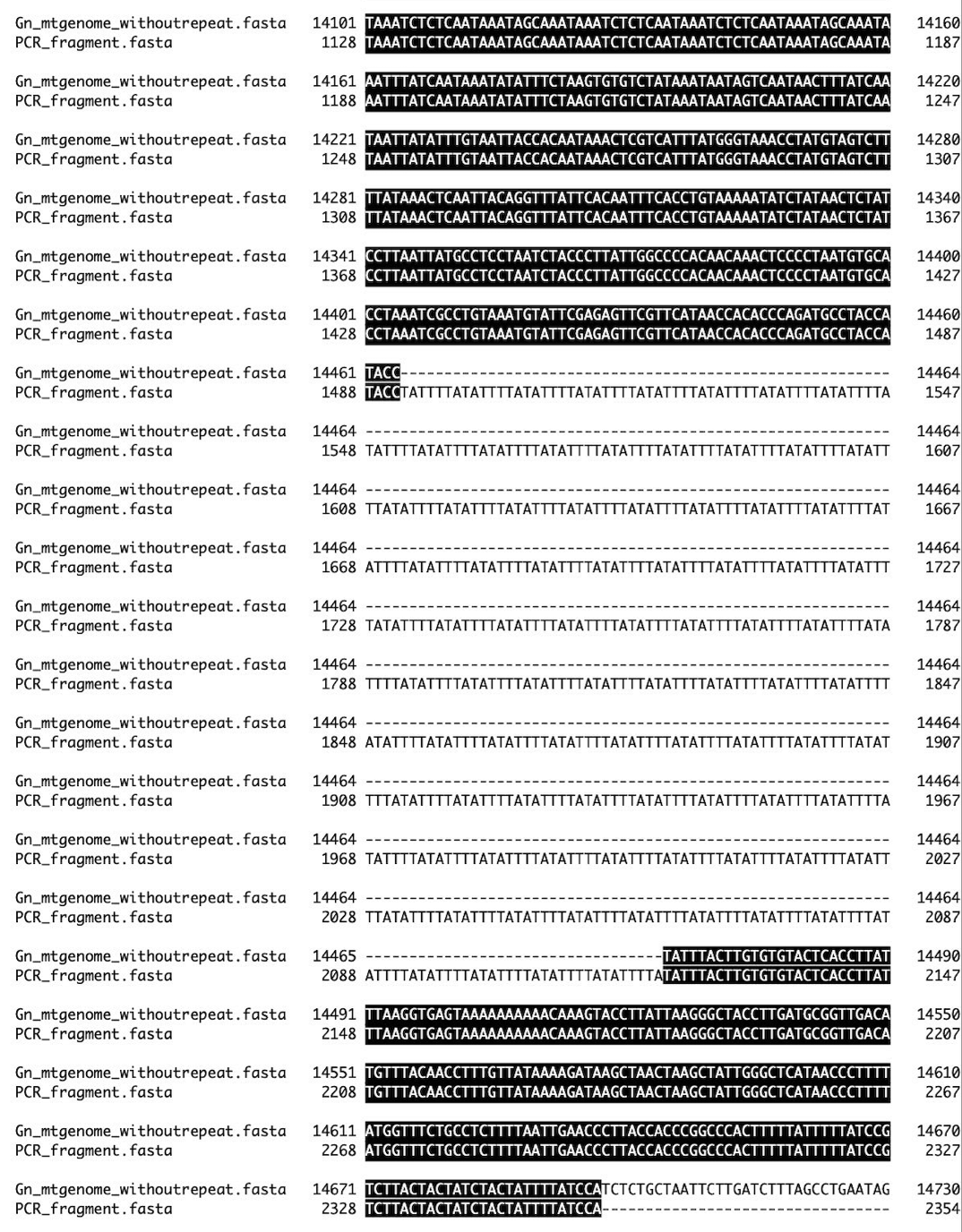


**Figure S2.** The alignment of the control region 2. The mitochondrial genome sequence assembled using the short read data is in the upper row, and the sequence of the PCR product of CR2 sequenced and assembled with MinION is the lower row.

**Reference:**

Thorvaldsdóttir H, Robinson JT, Mesirov JP (2013) Integrative Genomics Viewer (IGV): high-performance genomics data visualization and exploration. Briefings in Bioinformatics. 14(2):178-192. <https://doi.org/10.1093/bib/bbs017>.
